# Cycle Biodynamics of Women’s Microbiome in the Urinary and Reproductive Systems

**DOI:** 10.3390/jcm12124003

**Published:** 2023-06-12

**Authors:** Orly Grobeisen-Duque, Carlos Daniel Mora-Vargas, Ma. Guadalupe Aguilera-Arreola, Addy Cecilia Helguera-Repetto

**Affiliations:** 1 Departamento de Inmunobioquímica, Instituto Nacional de Perinatología Isidro Espinosa de los Reyes, Ciudad de Mexico 11000, Mexico; orly.grobeisen@gmail.com (O.G.-D.); mora.vargas.daniel@hotmail.com (C.D.M.-V.); 2 Facultad de Ciencias de la Salud, Universidad Anahuac Mexico, Ciudad de Mexico 52786, Mexico; 3 Escuela Nacional de Ciencias Biologicas del Instituto Politecnico Nacional, Ciudad de Mexico 11350, Mexico; marreoag@ipn.mx

**Keywords:** urinary microbiome, reproductive system microbiome, genitourinary microbiome, microbiota, infertility, reproductive stage, inflammation, dysbiosis, reproductive health

## Abstract

The genitourinary microbiome plays a crucial role in the establishment and maintenance of urinary and reproductive health in women throughout their lives. Particularly during the reproductive stage, resident microorganisms contribute to implantation and protect against perinatal complications, including preterm birth, stillbirth, and low birth weight, while also serving as the first line of defense against pathogens that can cause infections, such as urinary tract infections and bacterial vaginosis. This review aimed to elucidate the relationship between a healthy microbiome environment and women’s overall health. We examine the variability and dynamics of the microbiome during different developmental stages, ranging from the prepubertal to the postmenopausal stage. Furthermore, we explore the significance of a healthy microbiota in successful implantation and pregnancy development and investigate potential differences between women experiencing infertility. In addition, we analyze the local and systemic inflammatory responses associated with the establishment of a dysbiotic state and compare it to a condition where a healthy microbiome was established. Lastly, we present the most recent evidence regarding preventive measures, such as dietary interventions and the use of probiotics to promote and maintain a healthy microbiome, thereby ensuring comprehensive women’s health. By highlighting the importance of the genitourinary microbiome in reproductive health, this review aimed to enhance this microbiome’s visibility and significance in the field.

## 1. Introduction

The human microbiota is defined as the resident microorganisms, predominantly bacteria, that coexist with us in a symbiotic relationship. These microbial colonies residing within our bodies contribute to the establishment of a state of homeostasis, facilitating healthy human development. Furthermore, the microbiome encompasses all genomic information, genes, and microbial products generated by these resident microorganisms within the body [1].

In recent years, compelling evidence demonstrated the significant impact of the microbiome in promoting holistic health rather than merely preventing disease, considering humans as biopsychosocial beings. Several studies have explored the role of the gastrointestinal microbiome in synthesizing approximately 80% of the serotonin involved in neurobiochemical processes, leading to the emergence of a novel concept known as the microbiota–intestinal–brain axis. This axis is believed to play a crucial role in psychological and mental well-being [2]. 

Furthermore, diverse and rich microbiota that are high in *Lactobacillus* spp. exhibit anti-inflammatory and antipathogenic effects, promoting the establishment of ecological balance. In the social sciences, there has been an exploration of the theory emphasizing the significance of a well-balanced microbiome, demonstrating, in a reductionist manner, its essential role in the development of social relationships, skills, and affinity [2]. 

The Human Microbiome Project (HMP) was initiated by the National Institute of Health (NIH) as an endeavor to initially characterize the microbial populations inhabiting various body sites [3]. In this research, 48 primary areas were tested to identify the microbial composition and diversity. In complementary studies, the characteristics of each body site were determined, allowing us to understand the habitat conditions for different microorganisms. We found that the gastrointestinal tract, oral mucosa, nasal mucosa, and skin were among the most studied anatomical sites. Over the past few years, the number of studies researching the genitourinary microbiome in women has grown, highlighting its crucial role in reproductive and urinary health. Moreover, it is an integral part of the immune system, providing protection against pathogens that contribute to urinary tract infections (UTIs) and inflammatory processes that can lead to infertility [4,5].

In this review, we present the latest information on the characteristics of the genitourinary microbiome in women during reproductive age. We analyze the factors that determine its establishment and natural habitat. In addition, we present the hormonal mechanisms that impact bacterial survival within the genitourinary tract, recognizing that a state of dysbiosis can be the first step toward developing infectious and non-infectious diseases that affect women’s health during this particular phase of development. Finally, we review the effectiveness and availability of alternative and complementary immunomodulatory therapies for the establishment of a healthy microbiome.

## 2. Genitourinary Tract Microbiome

To understand the factors that promote the proliferation and establishment of the microbiome, it is necessary to define specific anatomical sites. The urinary tract can be divided into two parts: the upper part, which comprises the kidneys and ureters, and the lower part, which comprises the bladder and urethra. The female reproductive system consists of the vulva, cervix, uterus, uterine tubes, and ovaries. The genitourinary tract includes all the aforementioned components combined [3,6]. Throughout this review, we refer to the female reproductive system as either the menstruant or pregnant reproductive system, depending on the reproductive state of the woman.

**Figure 1:**
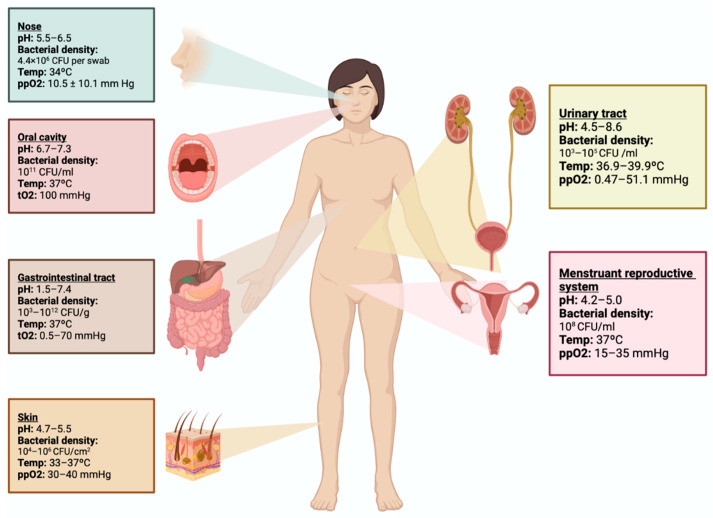
Anatomical site characteristics for microbiota establishment. The urinary tract and menstruant reproductive system, as well as the nose, oral cavity, gastrointestinal tract, and skin, possess distinct characteristics that support the development of resident microorganisms known as “microbiota”, which includes their genes, metabolism, and collective genome, which are referred to as the “microbiome”.

The urinary tract maintains a pH range of 4.5–8.6, a temperature between 36.9 and 39.9 °C, and an oxygen pressure of 0.47–51.5 mmHg. These conditions allow for the proliferation of bacteria, resulting in a microbial density ranging from 10^3^ to 10^5^ CFU per milliliter of urine. The menstruant reproductive system has a pH range of 4.2–5.0, a temperature of 37 °C, and an oxygen pressure of 15–35 mmHg, resulting in a microbial density of 10^8^ CFU per milliliter. The oxygen pressure of each tract supports the growth and proliferation of both aerobic and facultative anaerobic bacteria (Figure 1) [3,7]. These characteristics differentiate the capacity of a specific microbiota to inhabit specific anatomical sites. Among the microorganisms isolated from the genitourinary tract in healthy women of reproductive age, *Lactobacillus*, *Bifidobacterium*, and *Streptococcus* are the predominant families identified [8].

*Lactobacilli* are prominent in both the urinary tract and reproductive system [3]. Most *Lactobacillus* species produce lactic acid, which contributes to establishing an acidic pH and creating a favorable environment that helps to prevent viral, non-resident bacteria, and parasitic infections. Moreover, these microorganisms produce bacteriocins, which are proteins that exhibit antimicrobial properties and exert bactericidal effects, inhibiting bacterial growth. Some of these species also produce hydrogen peroxide, which acts as an additional antimicrobial agent. The presence of these various species facilitates the establishment of a healthy microbiome in the urinary tract and reproductive system, which is essential for the immune system’s defense against pathogens [5,9].

Among the *Lactobacillus* spp., *Lactobacillus iners*, which can be found in the urinary tract, does not produce lactic acid. If *L. iners* predominates, it may lead to an alkaline pH, which creates an ideal environment for the growth of uropathogens. In contrast, *Lactobacillus crispatus*, which is found in both the urinary tract and reproductive system, produces lactate, which helps to protect against bacterial vaginosis caused by *Chlamydia trachomatis* [9].

**Figure 2:**
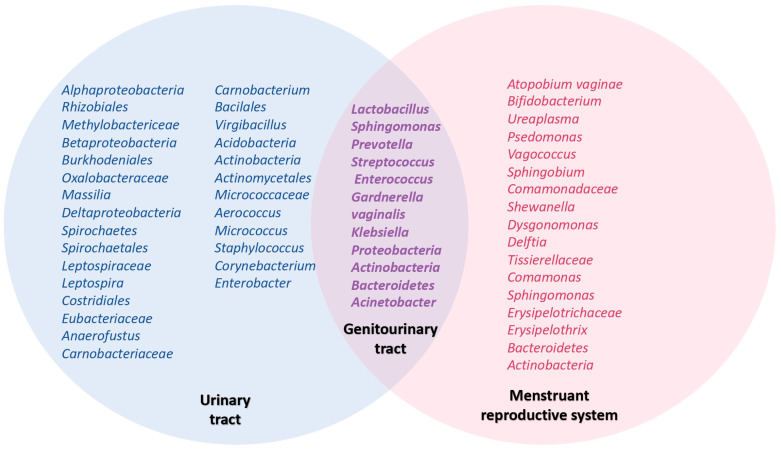
Venn diagram illustrating the resident microbiota in the urinary tract, menstruant reproductive system, and genitourinary tract. The distinctive characteristics of each anatomical site facilitate the normal and healthy development of microbiota. Shared microorganisms can be found in both the urinary tract and the menstruant reproductive system, which are collectively referred to as the genitourinary tract microbiota. This supports the theory that the vaginal microbiome serves as a reservoir of microbiota in cases of dysbiosis or isolated infections in the urinary tract.

A wide range of authors described the composition of the microbiome in the urinary tract and menstruant reproductive system, demonstrating that some bacteria are shared by both sites (Figure 2). Chen et al. examined the vagino-uterine microbiota composition in women of reproductive age, observing a higher microbial density of *Lactobacillus* spp. (97.56–99.99%) with increased richness and diversity in the menstruant reproductive system, ranging from the vulva to the cervix. In the upper sites, there was a reduced percentage of *Lactobacillus* spp. and a greater variety of other bacterial species.

Specifically, when examining bacteria isolated from the endometrium, 30.6% were *Lactobacillus* spp., 9.09% were *Pseudomonas*, 9.07% were *Acinetobacter*, and 7.29% were *Vagococcus*. In the uterine tubes, 18.27% were *Acinetobacter*, 11.49% were *Comamonas*, 9.9% were *Pseudomonas*, and 9.1% were *Pseudomonadaceae*. Finally, in the Douglas sac liquid obtained from these patients, 13.46% were *Pseudomonas*, 13.26% were *Vagococcus*, and 11.7% were *Acinetobacter* (Figure 2). These findings refute the notion that the bacteria of the microbiota are exclusively confined to the lower menstruating reproductive system, demonstrating that the microbiota can be found in both the upper and lower sites with high diversity and richness [6,10].

Regarding the urinary microbiota in women, Wolff et al. observed a high percentage of *Lactobacillus* spp., finding a higher proportion of *Lactobacillus crispatus*, *Lactobacillus jensenii*, and *Lactobacillus iners* [3,11]. Fouts et al. found a relative predominance of *Lactobacillus* spp. in urinary samples from healthy women in contrast to healthy male samples, where a higher proportion of *Corynebacterium* was observed [12]. Additionally, Siddiqui et al. studied the urinary tract and found a higher bacterial diversity in healthy women during their reproductive stage, with a higher abundance of *Lactobacillus* spp., followed by species of *Prevotella*, *Gardnerella*, *Peptoniphilus*, *Dialister*, *Finegoldia*, *Anaerococcus*, *Allisonella*, *Streptococcus*, and *Staphylococcus* (Figure 2) [13].

A new theory has emerged over the last few years, establishing the vaginal microbiome as a reservoir for the urinary tract. During dysbiotic periods, the urinary tract becomes prone to UTIs by uropathogenic organisms. In this state, the link of the vaginal tract promotes the recolonization of the resident bacteria, leading to less severe symptomatology and a shortening of the pathogenic period.

## 3. Genitourinary Microbiome during a Woman’s Lifetime

Many changes occur during the growth and development of women at different levels, including physiological, hormonal, anatomical, and biochemical levels. Adolescence is considered the transitional period between childhood and adulthood in which the body develops secondary sexual characteristics and obtains complete maturity of the different organs and systems [14]. In the different phases of the reproductive stage, women develop a diverse microbiome that allows for the establishment of a healthy urinary tract and reproductive system protected against pathogens.

**Figure 3:**
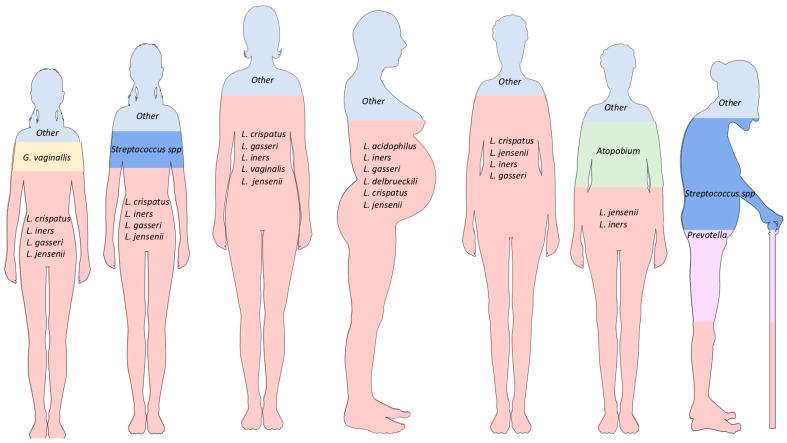
Lifetime changes in women’s microbiota proportions. Due to changes in anatomy, physiology, and hormones during different life stages, women experience an evolution of resident microorganisms, creating a healthy environment during fertile stages. At the extremes of life, there is higher diversity in the microbiota, but some species can lead to the development of genitourinary conditions and proinflammatory states. The high levels of estrogen during the reproductive stage promote the healthy development of *Lactobacillus* spp., allowing for a balanced environment and protection against pathogens. During pregnancy, the dynamicity resulting from hormonal and anatomical changes promotes the growth of *Lactobacillus* spp. and other bacteria. The increasing diversity of *Lactobacillus* spp., including *L. crispatus* and *L. jensenii*, may protect the mother–child dyad during the perinatal period, preventing complications and providing nourishment during the gestational period.

Hickey et al. showed an increase in the variety of *Lactobacillus* spp. in the vaginal and vulvar microbiomes during adolescence, comparing the microbiome found at the onset of menarche up to the middle stages of adolescence. During this period, there was more proliferation of *Lactobacillus crispatus*, *Lactobacillus iners*, *Lactobacillus gasseri*, *Lactobacillus jensenii*, and *Streptococcus* spp. [3,15]. In the pubertal period, there was also higher vaginal isolation of *Gardnerella vaginalis* in asymptomatic patients without an active sexual life, establishing it as a transitional resident microorganism in some women (Figure 3) [15]. 

In another study, Storm et al. analyzed and compared the urinary tract and reproductive system microbiomes of prepubertal girls who were not yet toilet trained and those in adolescence. The results showed a *Lactobacillus*-predominated environment in the urethra and vagina during adolescence, with a higher variety of species in the urethra compared with the prepubertal stage. The species found were *Lactobacillus gasseri*, *Lactobacillus iners*, and *Lactobacillus jensenii* (Figure 3). In contrast, prepubertal girls had an environment that was higher in *Prevotella*, with *Prevotella colorans* and *Prevotella timonensis* being the species with the highest abundances, along with other bacteria, such as *Dialister*, *Campylobacter*, and *Schaalia*. In the pre-toilet-trained girls, *Klebsiella* and *Enterococcus* were found to be common, with increased abundances of *Klebsiella oxytoca*, *Klebsiella pneumoniae*, and *Enterococcus hirae*. More research is needed to establish a correlation between genitourinary tract microbiome fluctuations in the prepubertal and adolescent stages [16].

As adults, women develop a healthy and diverse microbiome. M. Rostok et al. determined that the genitourinary microbiome of healthy women consists of *Lactobacillus* spp., including *Lactobacillus crispatus*, *Lactobacillus gasseri*, *Lactobacillus iners*, *Lactobacillus vaginalis*, and *Lactobacillus jensenii*. Specifically, *Lactobacillus crispatus* has been established as the species of *Lactobacillus* with the greatest abundance in a healthy microbiota (Figure 3) [10,17,18]. 

Saraf et al. proposed that women develop a dynamic environment throughout the body during the reproductive period, with predominance in the urinary tract and reproductive system. In this study, it was determined that more than 120 *Lactobacillus* spp. exist in the genitourinary microbiome, with greater importance found in *Lactobacillus crispatus* for its protective effect against pathogens [19].

During pregnancy, there are no significant changes in the skin, mouth, and gastrointestinal tract microbiota (Figure 3) [5]. Petricevic et al. concluded that there is a risk reduction of 75% for preterm birth in women with a greater abundance and diversity of *Lactobacillus* during the first trimester [9]. 

Furthermore, there is a decreased production of estrogens during menopause that causes vulvovaginal atrophy and alterations in the genitourinary microbiome. Saraf et al. proposed the possibility of a transitional microbiome at the end of the reproductive stage. They established that during the premenopausal period, a high quantity of *Lactobacillus* spp. may be found, with more significant proportions of *Lactobacillus crispatus*, *Lactobacillus jensenii*, *Lactobacillus iners*, and *Lactobacillus gasseri* (Figure 3). In perimenopausal women, a decrease in *Lactobacillus* spp. may be identified, with a higher prevalence of *Lactobacillus iners* and *Lactobacillus jensenii* (Figure 3). Additionally, an increase in other bacteria, such as *Atopobium* spp., may be found [19]. Finally, postmenopausal women have a higher proportion of *Streptococcus* and *Prevotella* species (Figure 3). Based on this information, an inverse association was established between the high quantity and diversity of *Lactobacillus* spp. in the vagina and the appearance of vaginal dryness, which is one of the main symptoms in postmenopausal women [20,21]. 

It is important to mention that there are differences in microbiome variety in different ethnic populations. In a study focused on the global ethnicity analysis of vaginal dysbiosis, Van de Wijgert et al. analyzed the composition of the vaginal microbiome during the reproductive stage in different ethnic groups residing in Amsterdam, the Netherlands. They found that women of sub-Saharan African descent had a greater proportion of Lactobacillus iners and less *Lactobacillus crispatus*, putting them at a greater risk for vaginal dysbiosis and infections, such as bacterial vaginosis, than Dutch women [22].

Ravel et al. conducted a study that examined the vaginal microbiome of different ethnic communities in North America, including White, Hispanic, Asian, and Black women. They found that 80.2% of Asian women and 89.7% of White women had a *Lactobacillus*-dominant environment characterized by a high abundance of *Lactobacillus crispatus*, *Lactobacillus gasseri*, *Lactobacillus iners*, and *Lactobacillus jensenii*. In contrast, only 59.6% of Hispanic women and 61.9% of Black women showed a *Lactobacillus*-rich microbiome. Both groups showed a higher vaginal pH (Hispanic 5.0 +/− 0.59 and Black 4.7 +/− 1.04), creating perfect conditions for the growth of non-dominant *Lactobacillus* communities with higher diversity, such as *Prevotella*, *Atopobium*, *Gardnerella*, *Sneathia*, *Megasphaera*, *Streptococcus*, *Atopobium*, and other strictly anaerobic bacteria [23].

Hernández-Rodríguez et al. studied pregnant Mexican women and established that they had a diminished quantity of *Lactobacillus* during the development of pregnancy, with a decrease in richness and diversity. This study found 78% *Lactobacillus acidophilus*, 54% *Lactobacillus iners*, 20% *Lactobacillus gasseri*, and 6% *Lactobacillus delbrueckii*, in contrast with European pregnant women who had 40% *Lactobacillus crispatus*, 27% *Lactobacillus iners*, 15% *Lactobacillus jensenii*, and 9% *Lactobacillus gasseri* [5].

## 4. Menstrual Cycle and Genitourinary Microbiome

**Figure 4:**
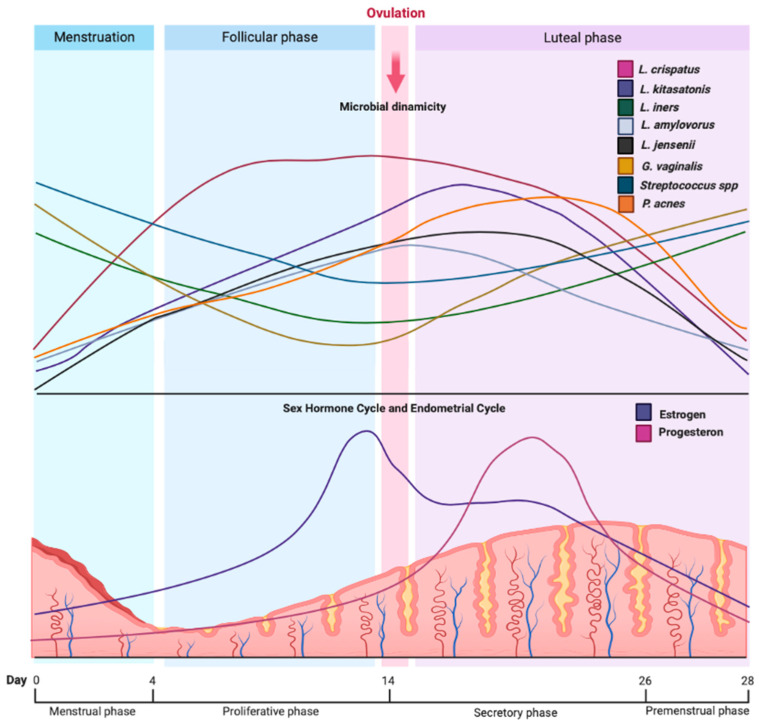
Microbial dynamics during the menstrual cycle. The diversity of bacteria during the menstrual cycle depends on variations in the sex hormone cycle (estrogen and progesterone) and endometrial fluctuations. In particular, during the stages with higher estradiol levels, species such as *L. crispatus*, *L. kitasatonis*, *L. amylovorus*, *L. jensenni*, and *P. acnes* increase their density in the genitourinary tract. Conversely, a decrease in estradiol and progesterone creates an environment rich in species such as *G. vaginalis*, *L. iners*, and *Streptococcus* spp., which can lead to infections and pro-inflammatory states. These changes depend on variations in the sex hormone cycle (estrogen and progesterone) and endometrial fluctuations during the different stages of the menstrual cycle.

During the menstrual cycle, hormonal and physiological changes can impact the diversity and richness of the genitourinary microbiome. Song et al. analyzed vaginal microbiota fluctuations across different stages of the menstrual cycle. The results reported a higher alpha diversity with a diminished relative abundance of *Lactobacillus* spp. and a higher proportion of other bacteria, such as *Streptococcus* spp., *Peptostreptococcus* spp., and *Anaerococcus* spp. [24]. Krog et al. showed that women have greater diversity in their vaginal microbiome during menstruation. Additionally, they found an increase in non-resident bacteria responsible for bacterial vaginosis, such as *Gardnerella vaginalis*, *Prevotella bivia*, *Ralstonia solanacearum*, *Staphylococcus aureus*, *Staphylococcus epidermidis*, *Ureaplasma parvum*, *Veillonella atypica*, and *Veillonella montpellierensis*. These bacteria were diminished during the follicular and luteal phases (Figure 4) [25]. 

Chaban et al. compared the microbiome during the different cycle phases (menstrual, follicular, preovulatory/ovulatory, and luteal), showing a higher diversity during the follicular phase than in the luteal phase [26]. In contrast, Krog et al. reported minimal changes in the microbiome during the transition from the follicular phase to the luteal phase and a higher variety at the beginning and during the menstrual period. Nevertheless, when comparing the follicular and luteal phases against the menstrual period, the study showed an increase in four *Lactobacillus* species in the genitourinary tract, namely, *Lactobacillus crispatus*, *Lactobacillus kitasatonis*, *Lactobacillus amylvorus*, and *Lactobacillus kefiranofaciens* during the first two phases (Figure 4) [25].

In another study, Chen et al. found that there was an increase in *Propionibacterium acnes* during the secretory phase at the endometrium (Figure 4). During this phase, there was also an increase in the microbial metabolism of porphyrin, arginine, and proline, as well as an increase in benzoate and nitrotoluene degradation and siderophore biosynthesis. In contrast, the proliferative phase showed an increase in resident bacterial growth, which was related to the high activation of metabolic pathways of pyrimidines and purines, as well as biosynthesis of aminoacyl-tRNA and peptidoglycans [6]. 

Hormonal analysis showed that changes in the microbiome are directly proportional to the estrogenic cycle, especially estradiol. High levels of this steroidal hormone increase the proliferation of *Lactobacillus* spp. This occurs due to an increased level of free glycogen in the vaginal mucosa derived from the activation of estrogen-linked mechanisms, leading to bacterial growth (Figure 4) [24]. Krog et al. presented an association between the levels of estradiol and an increase in *L. crispatus* proliferation, which acts with a proliferative effect during the follicular and ovulatory phases and with a slightly lower effect during the luteal phase. Nevertheless, high levels of estradiol show an increase in *Lactobacillus iners*, which could be a potential factor for establishing dysbiosis (Figure 4) [25].

To date, no conclusive studies have shown a specific role of progesterone concerning the genitourinary microbiome [25]. However, published studies have not shown any harmful effects on the resident microbiome or its diversity. Further research is needed to determine the impact of hormonal levels and the balance between estrogen and progesterone on the resident bacteria in the genitourinary tract, leading to an understanding of the endocrine–microbiota dynamics.

## 5. Hormonal Contraceptives and Genitourinary Microbiome

Different types of hormonal contraceptives available in the market can induce specific endocrine changes that can impact the ovarian and menstrual cycles. As mentioned previously, fluctuations in estrogen levels are associated with the diversity and richness of the genitourinary microbiota.

According to a study by Song et al., women using combined hormonal contraceptives experience more significant changes in their microbiome throughout different phases of the menstrual cycle, with a higher proportion of *Lactobacillus* spp. However, women using progesterone-only hormonal contraception showed fluctuations in their normal microbiome, which was characterized by a decreased proportion of *Lactobacillus* spp. compared with the combined group [24]. 

On the other hand, Krog et al. showed that women who did not use hormonal contraceptives experienced wide fluctuations in the genitourinary microbiome during the phases of the menstrual cycle in comparison with those who did. It was determined that the type of hormonal contraceptive had no significant association with the changes in the genitourinary microbiome compared with healthy women in their reproductive age who did not use it. The menstrual phase was the only period during the cycle that showed a significant difference in the genitourinary microbiome in women who did not use contraceptives [25].

## 6. Reproductive Health and the Genitourinary Microbiome

Microbiome diversity is essential to establish reproductive wellness and to develop a healthy gestational period. Changes in the genitourinary microbiome can lead to dysbiosis and the development of local and systemic inflammatory states, posing risks to women’s health during this stage of life.

### 6.1. Pregnancy

Pregnancy is characterized by several physicochemical changes that facilitate prenatal development. Fluctuating estrogen levels, a relative state of immunosuppression, and reduced mucosal barriers resulting from higher blood flow and vaginal mucosal edema all contribute to alterations in the urogenital microbiome. The imbalance of the microbiome in the genitourinary tract has been associated with adverse perinatal outcomes, including preterm birth, low birth weight, intrauterine infections, and stillbirth [27].

Aagaard et al. determined that pregnant women exhibit greater microbiome diversity and richness in their microbiome compared with non-pregnant women, with a predominance of *Lactobacillus*, mainly *Lactobacillus iners*, *Lactobacillus crispatus*, *Lactobacillus jensenii*, and *Lactobacillus johnsonii*. Pregnant women with a higher diversity of *Lactobacillus* spp. during the first trimester showed a risk reduction of 75% for perinatal complications compared with pregnant women with dysbiosis. Pregnant women with lower diversity and increased proportions of *Lactobacillus iners* showed an increased risk. Other bacteria identified in the genitourinary microbiome during pregnancy included *Clostridiales*, *Bacteroidales*, and *Actinomycetales* [28].

Furthermore, Cobo et al. characterized the microbiomes of the urinary and reproductive systems in pregnant women to establish a relationship with perinatal complications, such as preterm birth in patients with intraamniotic inflammation (IAI). The study determined that women with IAI exhibited greater microbial diversity but less bacterial richness. The study also established a relationship between a microbiome with a more significant proportion of *Lactobacillus* spp. in women without IAI, in contrast with women with IAI, who had substantial percentages of *Ureaplasma*, *Peptoniphilus*, *Gardnerella*, and *Haemophilus*. Even though a decreased amount of *Lactobacillus* spp. was seen in patients with IAI, normal levels of *Lactobacillus iners* were observed, establishing its association with preterm birth [29].

This study demonstrated that increased bacterial density and diversity of *Lactobacillus* spp. actively contributed to the development of chorioamnionitis. Conversely, it was established that women with less *Lactobacillus* diversity have a greater risk of preterm birth.

### 6.2. Infertility

Infertility can be defined as the incapacity to conceive after 12 months or more of regular unprotected sexual intercourse [30]. The theory that a healthy resident microbiome impacts reproductive health has recently been established. Studies showed that 19% of patients with infertility will suffer from concomitant bacterial vaginosis, while 39% will have genitourinary dysbiosis [19,31].

The bacteria found in the reproductive system during menstruation influence different stages of the reproductive process, such as gamete formation, fertilization, implantation, and pregnancy maintenance [32]. The presence of dysbiosis causes inflammation in the upper menstruant reproductive system, generating infertility [30,31]. One of the common causes related to infertility is endometriosis, which has recently been associated with the presence of *Actinomyces*, *Corynebacterium*, *Enterococcus*, *Escherichia coli*, *Fusobacterium*, *Gardnerella*, *Prevotella*, *Propionibacterium*, *Staphylococcus*, and *Streptococcus* in the genitourinary tract [6,31,33]. The presence of *Lactobacillus* spp. was shown to produce better reproductive and gestational results, especially with *Lactobacillus crispatus*, *Lactobacillus gasseri*, *Lactobacillus iners*, and *Lactobacillus jensenii* [9]. 

Wee et al. showed an increased proportion of *Ureaplasma* and *Gardnerella* compared with other bacteria found in the cervix and vagina in women with infertility. In another study, Moreno et al. observed an increase in *Gardnerella*, *Streptococcus*, and *Bifidobacterium* in patients with endometrial implantation problems [1,27]. Uterine infections caused by bacteria such as *Streptococcus* spp., *Staphylococcus* spp., *Enterococcus* spp., *Escherichia coli*, and *Klebsiella* pneumoniae increase the risk of infertility due to the activation of proinflammatory pathways in the menstruant reproductive system, causing problems with implantation [34]. Additionally, Riganelli et al. established a correlation between patients with infertility and implantation problems and a nondominant *Lactobacillus* environment in the genitourinary tract [33,35].

One of the treatments offered for infertility is in vitro fertilization (IVF). Research on this population showed increased dysbiosis in patients with implantation problems. Harr et al. reported high proportions of *Gardnerella vaginalis* and *Atopobium vaginae* among patients with infertility treated with IVF. An association was found in patients with tubal factor infertility with increased bacterial pathogens in the vaginal tract linked to bacterial vaginosis [27,36].

The studies mentioned above established a relationship between dysbiosis and different causes of menstruant reproductive system infertility, representing 35% of the grounds for couple infertility. The increased inflammation of the menstruant reproductive system, secondary to a diminished proportion of *Lactobacillus* spp. and increased non-resident bacteria in the genitourinary tract, represents a key tool to determine a proper and individualized diagnosis and treatment [37]. 

## 7. Urinary Tract Infections and Urinary Microbiome

The urinary tract is the site with a higher risk of bacterial infection in the body, with prevalence increasing in women, especially with advanced age and postmenopause. The most common uropathogens are *Uropathogenic Escherichia coli* (UPEC), *Klebsiella pneumoniae*, *Enterococcus faecalis*, and *Proteus mirabilis* [3,18,38,39].

The bladder is composed of urothelium in the lower urinary tract, which is a transitional epithelium covered by glycosaminoglycans (GAGs) [8]. Microbiome bacteria, such as *Lactobacillus*, *Bifidobacterium*, and *Streptococcus*, synthesize specific enzymes for GAG degradation into smaller glucose polymers, resulting in increased bacterial metabolism and the establishment of resident bacteria. Additionally, the urothelium has uroplakin, which is an intercellular protein that works as an anchor site for UPEC fimbriae type 1 [3].

Price et al. reported a difference in the microbiome found in healthy women compared with those with UTIs. The study determined that species such as *Klebsiella pneumoniae*, *Streptococcus agalactiae*, *Aerococcus urinae*, *Enterococcus faecalis*, *Escherichia coli*, *Staphylococcus aureus*, and *Streptococcus anginosus* were found more frequently and in greater abundance in patients with a UTI [39,40,41]. Moustafa et al. showed the existence of two microbial signatures in healthy patients, finding *Actinobacteria* and *Firmicutes* [39,42]. The presence of UPEC, *Klebsiella*, *Pseudomonas*, *Enterobacter*, *Acidovorax*, *Rhodanobacter*, and *Oligella* in the urinary tract was associated with patients suffering from a UTI [42].

Many studies showed that high levels of *Lactobacillus* spp. with high diversity, specifically with a high proportion of *Lactobacillus crispatus*, promote a healthy urinary tract by decreasing pH levels, thereby protecting against uropathogenic bacteria, especially UPEC [43]. In contrast, diminished diversity of the microbiome with high proportions of Lactobacillus iners was associated with a higher risk of a UTI due to alkalinization of the urinary tract in women of reproductive age [18,27,44].

During pregnancy, the reproductive system and urinary tract undergo physiological changes. At the 7th week of pregnancy, ureteral dilation occurs due to increased progesterone levels, promoting epithelial relaxation. From weeks 22–26, the fetus compresses the abdomen and pelvis, leading to hydronephrosis. Additionally, an increase in plasma volume decreases the urine concentration and bladder volume, generating a relative increase in the glomerular filtration rate. All of the aforementioned factors contribute to urinary stasis, renoureteral reflux, and biochemical changes in the urine (such as pH, osmolality, glucosuria, and aciduria), which promote an ideal environment for uropathogenic colonization [8,45].

These changes increase the risk of suffering from a UTI during pregnancy caused by Escherichia coli, Staphylococcus saprophyticus, Klebsiella spp., Enterobacter spp., Proteus spp., Enterococcus spp., and Streptococcus group B. Due to the pro-inflammatory pathways secondary to infection, 50% of the UTIs that occur during pregnancy will result in preterm birth. Of these infections, 2–15% will be asymptomatic. The lack of appropriate prenatal consultations and screening during pregnancy promotes the appearance of perinatal complications. Due to a lack of treatment, 30% of these asymptomatic UTIs will result in pyelonephritis, which is considered a high-risk pathology for the binomial [8]. 

The high diversity and abundance of *Lactobacillus* spp. in the urinary tract of pregnant patients, from conception to birth, were shown to be protective factors against UTIs. The diminished richness of the microbiome with increased species, such as *Lactobacillus delbrueckii*, *Lactobacillus gasseri*, and *Lactobacillus iners*, promotes the establishment of an ideal environment for uropathogenic bacteria and the generation of a proinflammatory habitat. This results in a higher risk of generating UTIs and associated complications [5].

## 8. Genitourinary Microbiome and Inflammatory Cascade 

During a women’s reproductive age, her microbiome functions as a part of the immune system by creating a protective environment against pathogens and regulating the body’s inflammatory processes However, with the development of dysbiosis and the appearance of pathogenic species, the microbiota–immune system axis becomes saturated, promoting local and systemic proinflammatory states.

The study conducted by Mtshali et al. showed an association between dysbiosis and the prevalence of bacteria known to cause bacterial vaginosis, such as *Gardnerella vaginalis*, in women in their reproductive stage from South Africa. Imbalances in the microbial environments can lead to an increase in serum levels of TNF-α, IL-1β, IL-8, and LIF, which establish a pro-inflammatory state. Conversely, an increased proportion of *Lactobacillus crispatus* in the genitourinary microbiome was linked to a higher production of the GM-CSF protein [4,33,46,47,48]. This suggests that the composition of the genitourinary microbiome plays a role in regulating the immune response and inflammatory state, with specific bacterial species having differing effects on immune markers and inflammation.

**Figure 5:**
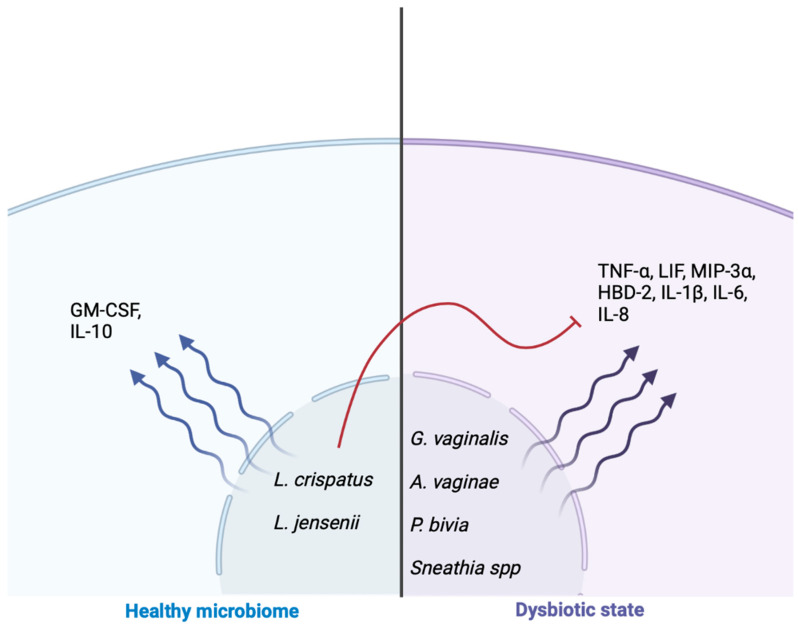
Inflammation states induced by cytokines as a product of a healthy microbiome and dysbiotic state. In a healthy microbiome, species such as *L. crispatus* and *L. jensenii* produce anti-inflammatory cytokines, such as GM-CSF and IL-10, which help to modulate the pro-inflammatory cytokines that develop during a dysbiotic state.

Doerflinger et al. determined that *Gardnerella vaginalis* secretes toxins, such as vaginolisine and sialidase, inducing the synthesis and release of cytokines, such as IL-8. Additionally, *Atopobium vaginae* induces the production of MIP-3α, HBD-2, IL-1β, IL-8, IL-6, and TNF-α, which are associated with proinflammatory damage of the urogenital epithelial barrier. *Prevotella bivia* exhibited increased production of proinflammatory cytokines at the genital level, which was inversely proportional to high levels of *Lactobacillus* spp. (Figure 5) [47,48,49,50,51].

Genitourinary tract *Lactobacillus* spp. have an anaerobic glucose metabolism that directly contributes to an anti-inflammatory effect by reducing the levels of cytokines, such as IL-6, IL-8, TNF-α, RANTES, and MIP-3α, which are secondary to the stimulation of TLR2, TLR3, and TLR4 agonists. *Lactobacillus crispatus*, in particular, is known for its anti-inflammatory properties. In an environment with low diversity and decreased levels of *Lactobacillus crispatus*, cytokine levels tend to increase (Figure 5). Certain bacteria, such as *Lactobacillus crispatus* and *Lactobacillus jensenni*, exhibit buffering characteristics in proinflammatory states [33,46].

Jean et al. conducted a study on pregnant women and found that the presence of *Lactobacillus crispatus* in the genitourinary tract was associated with a decrease in the release of cytokines, including IL-12, IL-17, MIP1-α, and VEGF. Additionally, women with bacteria associated with bacterial vaginosis, such as *Sneathia* and *Gardnerella vaginalis*, exhibited reduced levels of G-CSF and increased levels of IL-9, IL-10, and TNF-α [32,46] (Figure 5). Although cytokines and lipid mediators play an essential role in maintaining pregnancy and the inflammatory processes necessary for labor, the activation of proinflammatory pathways secondary to bacterial vaginosis with the release of cytokines, such as IL-1β, IL-6, IL-8, and TNF-α, increases the risk of presenting complications, such as preterm birth [52].

## 9. Probiotics and Diet for the Establishment of the Genitourinary Microbiome

The recent interest in studying the genitourinary microbiome in women of reproductive age has led to theories suggesting that resident microbiota act as a defense mechanism against pathogenic bacteria. Therefore, promoting a healthy microbiome and preventing dysbiosis may help to protect against pathogenic processes. Researchers have been exploring the potential use of probiotics and lifestyle changes, particularly dietary modifications, to restore and maintain a healthy genitourinary microbiome and its protective effects against uropathogens and bacterial vaginosis-causing agents [33].

Wolff et al. observed that administering an oral capsule twice a day containing *Lactobacillus rhamnosus* and *Lactobacillus reuteri* (109 UFC/mL) did not result in significant changes in the diversity of the urinary tract microbiome. However, the study showed that probiotics significantly reduce yeasts and coliforms in the genitourinary tract, improving the restoration of a *Lactobacillus*-dominant microbiota after bacterial vaginosis [11]. Rostok et al. conducted a study on healthy women of reproductive age, which demonstrated that the oral intake of probiotics containing *Lactobacillus crispatus* resulted in an average reduction in the concentration of *Gardnerella vaginalis* in the genitourinary tract. The authors concluded that administering this treatment in 7-day cycles resulted in an improved state of vaginal microbiota [17]. 

Meanwhile, Husain et al. determined that the oral administration of *Lactobacillus rhamnosus* and *Lactobacillus reuteri* capsules during the first trimester of pregnancy did not affect the prevalence of bacterial vaginosis or the vaginal microbiota composition between weeks 18 and 20 of gestation [53]. On the other hand, Riganelli et al. assessed the response of patients with infertility looking for IVF therapy, showing vaginal and endometrial microbiome restoration using combined treatment with probiotics and hormones, resulting in a favorable state of implantation [35].

Studies performed on the Asian population by Liang et al. reported that oral administration of probiotics containing *Bifidobacterium longum*, *Lactobacillus delbrueckii*, and *Streptococcus thermophilus* did not increase the risk of perinatal complications, such as preterm birth. They observed a relative increase in *Ureaplasma* and *Gardnerella* without an increased risk of this complication. Additionally, there was an increased concentration of *Veillonella* in the genitourinary tract, resulting in increased lactate production from the bacteria found in the probiotics, creating a high-carbon environment for the growth and proliferation of these bacteria [54].

Regarding diet, Song et al. did not find a significant relationship between the specific intake of nutrients, sugars, fiber, protein, and fats and the establishment of a genitourinary microbiome. On the other hand, other studies showed that a diet rich in folate, vitamin A, and calcium provides a protective factor against bacterial vaginosis. Meanwhile, diets high in fats are associated with an increased risk [24]. Additionally, David et al. established that a diet with a low intake of micronutrients, such as vitamins A, C, E, and D, folate, calcium, and β-carotene, increases the risk for bacterial vaginosis secondary to dysbiosis [48,55,56].

The hypercaloric and hyperlipidic Western diet increases the risk of genitourinary dysbiosis, creating an ideal environment for pathogen colonization in the reproductive system of pregnant and menstruating women. There is a hypothesis that a diet with high levels of starch promotes the presence of glycogen at the vaginal level, allowing for a higher concentration of *Lactobacillus* spp. due to the metabolism of *Lactobacillus* where free glycogen gets converted into lactate. Nevertheless, the studies have yet to be conclusive in their results. From the analyzed published articles, it was proposed that a diet high in fat and starch and low in fiber results in a higher risk of genitourinary microbiome alterations [55].

In pregnant women, Sun et al. determined that the microbiome of women who ate whole-grain cereals showed a more stable microbiome with less diversity, in contrast with the patients who ate hyper-processed grains. They also showed decreased concentrations of *Clostridiales*, such as *Ezakiella* and *Peptoniphilus* [57]. Additionally, Chavarro et al. established a relationship between the increased consumption of animal protein and a higher risk of developing genitourinary dysbiosis, which increases the prevalence of bacterial vaginosis and infertility [58]. Akoh et al. performed a study on adolescent pregnant women, establishing that low plasma levels of vitamin D were associated with a high risk of presenting bacterial vaginosis and increasing the concentrations of TNF-α [56].

Information regarding the benefits of consuming probiotics during the reproductive stage to maintain a healthy genitourinary microbiome is still controversial, showing a higher impact on the pregnant and menstrual reproductive systems. Regarding establishing a nutritional regimen, the studies reflect the necessity of taking care of vitamin and mineral plasma levels in women at their reproductive ages to develop a better balance and diversity of the genitourinary tract microbiome, aiming to avoid bacterial vaginosis. High-fiber diets with a balance between animal and vegetable proteins and low in saturated fats with complex carbohydrates (cereals, fruits, and vegetables) provide a beginning for establishing a healthy microbiota in the genitourinary tract.

The management of urinary tract infections conventionally involves the use of antibiotics as first-line treatment. Numerous studies attempted to identify alternative therapies to address dysbiosis and infections in the urinary tract, aiming to promote the establishment of a balanced and healthy microbiome. The utilization of immunomodulatory interventions offers the potential for targeted and individualized treatments, thereby reducing the adverse effects associated with indiscriminate antibiotic use for all genito-urinary infections [59].

The exploration and investigation of alternative immunomodulatory therapies, such as cranberry extract (which increases the Tamm–Horsfall glycoprotein to inhibit bacterial adhesion) [60,61,62,63], vitamin C and anthocyanins (which inhibit reactive oxygen species production to prevent urinary infections) [64,65,66,67], dietary restriction of iron, and lactoferrin supplementation (which reduces bacterial density in the urinary tract, particularly in cases with siderophore characteristics) [68,69], and subcutaneous insulin injections in diabetic patients (to promote the secretion of RNase 7, RNase4, and lipocalin-2 to prevent cystitis) [70,71], as well as vaccines [72,73] and probiotics [52], present promising avenues for future medical advancements. However, studies that investigated the use of immunomodulatory therapies as alternatives to antibiotics currently lack clinical significance, leading to a debate on the use and applications of these therapies. Further research beyond animal models is necessary to establish a meaningful correlation, requiring studies with greater statistical power.

## 10. Conclusions

In conclusion, the traditional belief that the reproductive system and urinary tract are sterile was challenged by recent research, which showed the presence of resident microorganisms in these areas, promoting symbiotic health. As was mentioned in the text, the acidic pHs of the urinary tract (4.5–8.6) and the menstruant reproductive system (4.2–5), along with the oxygen pressures of 0.47–51.5 mmHg and 15–35 mmHg, respectively, facilitate the growth and predominance of a microbiome rich *in Lactobacillus* spp., thus preventing the development of pathological conditions.

During the reproductive stage, women experience cyclical and physicochemical changes, requiring a body–microbiome balance to be protected against pathogens and establish complete health. Many factors are involved in establishing a microbiome, such as diet, hormonal levels, reproductive dynamicity, and antibiotic and probiotic consumption. The dynamics of estrogen levels during the menstrual cycle provide insight into how hormones affect the colonization of the genitourinary tract microbiome. In the early phases of the menstrual cycle, the microbiome is rich and has a high density of *Lactobacillus* spp. However, during menstruation, the microbial diversity decreases, leading to the establishment of a dysbiotic state. By promoting microbial health through resident bacteria, it becomes possible to establish a comprehensive biological balance that impacts women’s development and well-being at different stages of life. 

Dysbiosis of the genitourinary tract can lead to complications that directly affect women’s health. The establishment of urinary tract infections, bacterial vaginosis, infertility, stillbirth, and preterm birth was associated with imbalances in the microbiota. Understanding the correlation between the microbiome and its role in defense against pathogens and proinflammatory states provides new opportunities for preventive and therapeutic approaches in diagnosing and treating women’s health issues.

Although there are many probiotic products available on the market, both for vaginal and oral use, further research is needed to establish comprehensive therapies that promote reproductive and urinary health. There are numerous opportunities to explore immunomodulatory therapies as alternative and complementary measures, given the need for conclusive evidence and clinical significance in maintaining or restoring the genitourinary microbiota. More conclusive evidence is required to determine the relationship between immunomodulatory therapies and the maintenance or restoration of a healthy genitourinary microbiota.

## Data Availability

Not applicable.
